# Affective video recommender systems: A survey

**DOI:** 10.3389/fnins.2022.984404

**Published:** 2022-08-26

**Authors:** Dandan Wang, Xiaoming Zhao

**Affiliations:** Department of Computer Science, Taizhou University, Taizhou, China

**Keywords:** video recommendation, affective video recommender system, multidiscipline, multimodal, neuroscience, affective analysis, physiological signals, deep learning

## Abstract

Traditional video recommendation provides the viewers with customized media content according to their historical records (e.g., ratings, reviews). However, such systems tend to generate terrible results if the data is insufficient, which leads to a cold-start problem. An affective video recommender system (AVRS) is a multidiscipline and multimodal human-robot interaction (HRI) system, and it incorporates physical, physiological, neuroscience, and computer science subjects and multimedia resources, including text, audio, and video. As a promising research domain, AVRS employs advanced affective analysis technologies in video resources; therefore, it can solve the cold-start problem. In AVRS, the viewers’ emotional responses can be obtained from various techniques, including physical signals (e.g., facial expression, gestures, and speech) and internal signals (e.g., physiological signals). The changes in these signals can be detected when the viewers face specific situations. The physiological signals are a response to central and autonomic nervous systems and are mostly involuntarily activated, which cannot be easily controlled. Therefore, it is suitable for reliable emotion analysis. The physical signals can be recorded by a webcam or recorder. In contrast, the physiological signals can be collected by various equipment, e.g., psychophysiological heart rate (HR) signals calculated by echocardiogram (ECG), electro-dermal activity (EDA), and brain activity (GA) from electroencephalography (EEG) signals, skin conductance response (SCR) by a galvanic skin response (GSR), and photoplethysmography (PPG) estimating users’ pulse. This survey aims to provide a comprehensive overview of the AVRS domain. To analyze the recent efforts in the field of affective video recommendation, we collected 92 relevant published articles from Google Scholar and summarized the articles and their key findings. In this survey, we feature these articles concerning AVRS from different perspectives, including various traditional recommendation algorithms and advanced deep learning-based algorithms, the commonly used affective video recommendation databases, audience response categories, and evaluation methods. Finally, we conclude the challenge of AVRS and provide the potential future research directions.

## Introduction

Emotion or affection is a mental state which is unconscious and spontaneously arises accompanied by physiological and psychological status changes in human organs and tissues, e.g., heart rate, facial expression, brain, etc. (Shu et al., 2018b). Emotions are universal and have proved to be a highly multidisciplinary research field, from psychology, sociology, and neuroscience to computer science (Baveye et al., 2018). The emotional state of a consumer determines his behavior and decision-making process, i.e., click, purchase, or close. However, the viewer’s emotional state is ignored in the recommendation process because of the complexity of the mutual interaction of physiological signals with human emotions. The subtle emotional expression is straightforward to be misunderstood. Previous studies have mainly focused on users’ affection by ratings (Roy and Guntuku, 2016), comments (Orellana-Rodriguez et al., 2015), helpfulness votes, etc. However, acquiring this feedback requires users’ cooperation, and some require plenty of time. Therefore, the amount of such feedback data is limited and faced with a cold-start problem.

Recent research employs techniques closely related to neuroscience and human-robot interaction (HRI). The viewers’ emotional states are obtained from analyzing their physical and internal signal parameters with the help of various equipment. For example, researchers apply photoplethysmography (PPG) to estimate users’ pulse by using the fluctuations in skin color related to blood volume and the proportion of reflected light (Bohlin et al., 2019). Dabas et al. (2018) studied human emotions with the help of electroencephalogram (EEG) signals. De Pessemier et al. (2019) facilitated HRI for users to watch videos by an automated procedure based on facial recognition. The automatic feedback is gathered when users play the videos using a front-facing camera. The viewer’s physiological data is easy to get and can be obtained by several methods without the user’s active cooperation in the viewing process. The physiological data can be achieved by measuring body parameters, including skin estimated pulse, heart rate, mood, motion, shot change rate, and sound energy. The viewers’ psychophysiological signals of heart rate (HR) were calculated from an echocardiogram (ECG), while electro-dermal activity (EDA) and brain activity (BA) in EEG signals (Ðorđević Čegar et al., 2020). Facial expressions or features can be obtained by a camera (Tkalčič et al., 2013a).

The affective computing technology promotes the rapid development of the affective video recommender systems (AVRSs). An AVRS is a new trending research direction of recommender families in recent years. Unlike text, image, and speech emotion recognition (Zhang et al., 2022), AVRS mainly analyzes the emotional states in videos and detects emotional reactions according to different scenes. An AVRS recommends video resources that viewers may be interested in based on the recognized emotional states. As a new branch of affective analysis and recommender systems, it is necessary to define AVRS according to previous literature research.

Definition 1: AVRS: is a multidiscipline and multimodal HRI system that videos are recommended based on the reviewers’ emotional responses (implicit or explicit), e.g., physical, physiological signals, comments, etc.

The physical data reflect communicative signals, e.g., facial expressions, speech detection, and eye-tracking while viewing the video (Lim et al., 2020). In contrast, the physiological signals record body variations, e.g., heart rate, temperature, and blood pressure changes. These physical and physiological signals and comments are recognized and interpreted into emotional states. The AVRS recommends the videos based on emotion models according to the viewers’ emotional states.

### The differences between this survey and former studies

An AVRS is a relatively new recommender family branch that has begun to develop in recent years. At present, there are few comprehensive reviews related to affective video recommendations. Most works mainly focus on different domains of recommender systems, including recommender systems (Singh et al., 2021), the application of deep learning in recommender systems (Guo et al., 2017), tourism recommendation systems based on emotion recognition (Santamaria-Granados et al., 2021), affective recommender system techniques (Raheem and Ali, 2020), etc.

As shown in Table 1, we compare different aspects of our survey and recently existing related reviews, i.e., multimodal feature, multimodal data sources, deep learning methods, affective computing, multidiscipline knowledge, and video contents. Singh et al. (2021) mainly focused on different recommendation methods and existing problems without involving multimodal features, multimodal data sources, and multidiscipline knowledge. Zhang et al. (2019) provided a review of deep learning-based recommendations. However, they failed to supply multimodal data sources, affective computing, and multidiscipline knowledge. In Santamaria-Granados et al. (2021), they explored the emotional recognition of recommender systems in the tourist scenario. They provided guidelines for establishing emotion-sensitive tourist recommender systems. Unfortunately, they only cover a few publications related to multimodal data sources and video content. The contribution of Raheem and Ali (2020) is one of very few research works in the field of affective recommendation; they introduced the application of recommendation technology based on affective computing. However, Raheem and Ali (2020) haven’t explored multimodal data sources and multidiscipline knowledge. This survey aims to provide a comprehensive review of current research on AVRS, to discuss the open problems and limitations, and point out future possible directions.

**TABLE 1:** Comparisons between this survey and existing reviews.

Main concerns	Singh et al. (2021)	Zhang et al. (2019)	Santamaria-Granados et al. (2021)	Raheem and Ali (2020)	Our survey
Multimodal feature	×	✓	✓	✓	✓
Multimodal data sources	×	×	Few	×	✓
Deep learning methods	✓	✓	✓	✓	✓
Affective computing	✓	×	✓	✓	✓
Multidiscipline knowledge	×	×	✓	×	✓
Video content	✓	✓	Few	✓	✓

### The method of collecting relevant publications and the distribution

The relevant publications in this survey are obtained from Google scholar and published by Science Direct, Springer, IEEE, ACM, etc. The collected publications are from 2009 to 2022; filters are applied to the search engine by subject (affection, emotion, sentiment, affective computing, video recommendation, recommender systems). Table 2 illustrates the number of publications and the percentage from different sources.

**TABLE 2:** Publications from different sources.

Databases	Number of publications	Percentage
ACM	11	11.96%
IEEE	35	38.04%
Elsevier	11	11.96%
Springer	15	16.30%
Others	20	21.74%
Total	92	100%

We collected 92 non-repeated publications related to AVRS. Most of the articles are from IEEE, accounting for 38.04%, more than three times that of ACM and Elsevier. The distribution of publications from ACM, Elsevier, and Springer is similar, accounting for about 11–17%. The remaining publications are from various published websites. It can be seen from Table 2 that the number of publications related to AVRS is relatively limited compared with other fields of recommender systems, and it is thus in its infancy, which requires a large number of researchers and their outstanding work.

**FIGURE 1:**
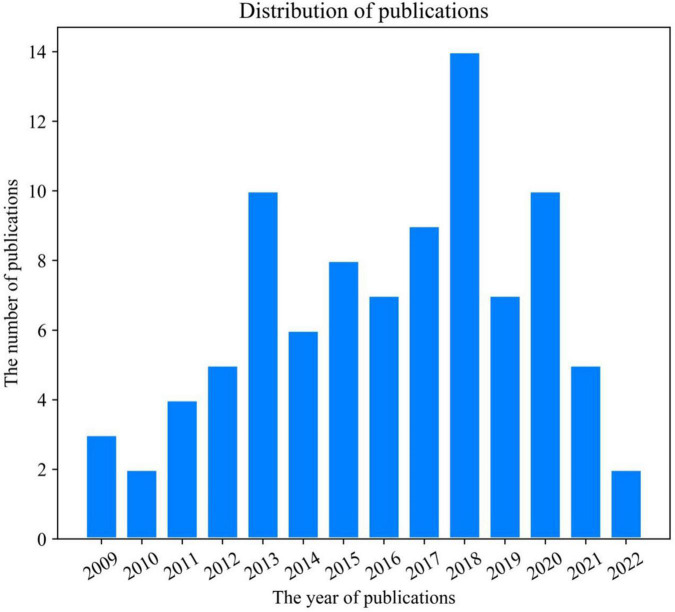
Distribution of publications in AVRS.

The distribution of AVRS publications is shown in Figure 1. The x-axis represents the year of publication, and the y-axis represents the total number of publications in the corresponding year. As we can see from Figure 1, the number of research works on AVRS is scarce. Since the relevant articles were published in 2009, there have been no more than ten published articles every year except in 2018, reaching the peak of 14 in 2018 and showing an apparent downward trend afterward. The publication distribution in Figure 1 also indicates that the prosperity of AVRS currently requires a great deal of academic dedication.

### Contributions of this survey

This survey provides a concise, comprehensive understanding of the latest AVRS research and gives dynamic guidelines in AVRS for scientific researchers, practitioners, and developers interested in video recommendations. We define the internal logic and operating mechanism of various models and algorithms, the classification of existing technologies and their characteristics, the databases for affective computing, the types of audience responses, and the evaluation metrics. The main contributions of this survey are summarized in the following three aspects:

(1) We systematically summarize and overview the current techniques in the affective video recommendation field.
(2) We classified the works of literature related to different models and algorithms, the possible database resources for video recommendation, the types of audience responses, and the evaluation metrics.
(3) We show the current challenges in the video recommendation field and envision possible future research directions.

The structure of this survey is arranged in the following: Section 2 introduces currently-used algorithms and models of video recommender systems; Section 3 shows the database resources commonly used in the research of AVRS; Section 4 classifies the ways to obtain user responses in publications; Section 5 summarizes the evaluation metrics of recommendation effect in different publications; Section 6 analyzes the challenges in the current research and discusses future research directions.

## The state-of-the-art affective video recommendation algorithms and models

**FIGURE 2:**
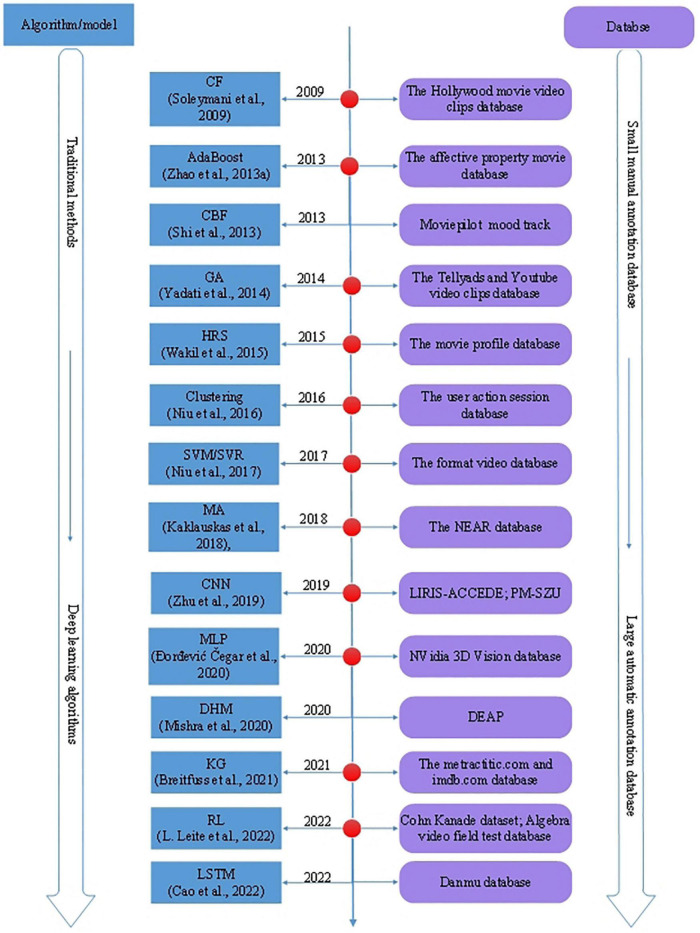
The evolution of AVRS with different algorithms and databases.

Video recommendation is based on video features and the viewers’ profiles. According to video clips, the viewers’ emotions are challenging to be captured simultaneously. Therefore, an AVRS is a more complex domain in recommender systems. Several researchers tend to solve the AVRS problem by various methods, traditional models, or algorithms, including support vector machine/support vector regression (SVM/SVR) (Arapakis et al., 2009a), clustering (Song and Yang, 2022), AdaBoost (Zhao et al., 2013), matrix-based algorithm (MA) (Kaklauskas et al., 2018), collaborative filtering (CF) (Diaz et al., 2018), content-based filtering (CBF) (Deldjoo et al., 2018), knowledge graph (KG) (Breitfuss et al., 2021), genetic algorithms (GA) (Wang and Chen, 2020), hybrid recommendation systems (HRS) (Wakil et al., 2015), the combination of several traditional recommendation algorithms, etc. Deep learning (DL) has gradually penetrated the field of affective computing and promoted the development of video recommendations. Deep learning-based models applied in AVRS in recent years include reinforcement learning (RL) (Leite et al., 2022), convolutional neural network (CNN) (Zhu et al., 2019), long short-term memory (LSTM) (Cao et al., 2022), multilayer perception (Ðorđević Čegar et al., 2020) (MLP), deep hybrid models (DHM) (Mishra et al., 2020), etc. The evolution of AVRS with different algorithms and databases is illustrated in Figure 2.

Table 3 shows the publications of this survey based on different techniques. From Table 3, we can infer that the most general approaches used for video recommendation are SVM/SVR, MA, CF, and CBF methods. Other models adopted in AVRS are relatively rare, especially the work of deep learning-based algorithms.

**TABLE 3:** Publications based on different techniques.

Categories	Algorithm/Model	Publications
Traditional methods	SVM/SVR	Arapakis et al., 2009a,b; Soleymani and Pantic, 2012; Soleymani et al., 2012; Srivastava and Roy, 2014; Sivakumar et al., 2015; Niu et al., 2017; Dabas et al., 2018; Bohlin et al., 2019
	Clustering	Niu et al., 2013; Niu et al., 2016
	AdaBoost	Zhao et al., 2013; Shu et al., 2018a
	MA	Tkalčič et al., 2013b; Dnodxvndv et al., 2018; Kaklauskas et al., 2018
	CF	Soleymani et al., 2009; Winoto and Tang, 2010; Tkalčič et al., 2013b; Choi et al., 2016; Diaz et al., 2018
	CBF	Shi et al., 2013; Tkalčič et al., 2013b; Deldjoo et al., 2018
	KG	Breitfuss et al., 2021; Qi et al., 2021
	GA	Yadati et al., 2014
	HRS	Mugellini et al., 2014; Wakil et al., 2015
Deep learning-based methods	RL	Tripathi et al., 2018; Leite et al., 2022
	CNN	Hewitt and Gunes, 2018; Kwon et al., 2018; Yang et al., 2019; Zhu et al., 2019
	LSTM	Alhagry, 2017; Zhang and Zhang, 2017; Ogawa et al., 2018; Nie et al., 2020; Wang et al., 2020; Cao et al., 2022
	MLP	Boughrara et al., 2016; Ðorđević Čegar et al., 2020; Krishnamurthy, 2020
	DHM	Fan et al., 2016; Liu et al., 2017; Yenter, 2017; Zhang et al., 2018; Tripathi et al., 2019; Mishra et al., 2020

In this section, we classify the publications according to the adopted algorithms or models. We first introduce several commonly-used traditional video recommendation algorithms, then describe the application of prevalent deep learning algorithms in AVRS, and analyze the advantages and disadvantages of both conventional recommendation algorithms and deep learning algorithms.

### Traditional methods

#### Support vector machine (SVM) or support vector regression (SVR)

The fundamental idea of implementing SVM and SVR is classifying the mixed input features to predict the users’ emotional states during their interaction with the robots. An SVM/SVR is one of the most widely-used techniques in the affective video recommendation domain. Researchers devoted valuable efforts to promoting the performances of video recommendations based on SVM/SVR. In Arapakis et al. (2009a), they trained a two-layer hierarchical SVM model by using interactive data, context information, and user response to determine whether the user’s unknown video is relevant or not. In Bohlin et al. (2019), they a support vector classifier was used to predict the ratings of video viewers and whether they will watch similar videos.

In Arapakis et al. (2009b), they leveraged a two-layer hierarchical SVM model to discriminate whether the video is relevant to a user. The real-time facial expressions were adopted for constructing a face model and classified into seven emotion categories. The classification results were forwarded to an SVM model to determine whether the videos were relevant or not. In Dabas et al. (2018), the authors classified users’ emotions when watching musical videos by constructing a 3D emotional model consisting of several octants including eight emotional states, i.e., relaxed, peaceful, bored, disgusted, nervous, sad, surprised, and excited. The human emotions were studied using EEG signals on the DEAP database (Soleymani et al., 2012). In Soleymani et al. (2012), they proposed a facial expression recognition algorithm. In particular, they first extracted frames from video sequences. Then, the structures were used to locate the faces, and a feature extractor was employed to extract face features. Finally, the extracted face features were normalized to obtain a higher level feature set, followed by training the SVM classifier to recognize facial expressions in real-time. A modality fusion strategy with an SVM (Soleymani and Pantic, 2012) was used to classify arousal and valence into three categories, respectively. The SVM with RBF kernel was utilized to identify the samples by discriminative features from two modalities. However, the problem with employing an SVM in a fusion scheme is that the output of SVM classifiers is uncalibrated; it is not directly usable, being a confidence value when combining results of different classifiers. Therefore, in Soleymani and Pantic (2012) they used two methods to tackle the problem, i.e., to model the probability of two classes determining the output values of SVM and adopting a solution to the extent of multiple courses.

Although these SVM-based algorithms have made significant progress in affective video recommendation, they are facing the problem of ignoring the temporal video factor and seriously affecting the recommendation quality. To solve this problem, Niu et al. (2017) studied the temporal element of emotion, i.e., the characteristics of emotional fluctuation. They proposed a method based on Grey Relational Analysis (GRA) to solve the above-mentioned problems. First, video features were extracted and mapped to Lovheim emotion space through an SVM. Then, GRA calculated the relationship between videos based on emotional features. Finally, the Fisher model was used for video recommendation, and their method proved effective when recommending temporal video sources.

In Srivastava and Roy (2014), they used an SVR to extract the connotative features of the movie’s audio to represent user reaction impressions. The SVR ranked the film according to the connotative features and then compared the ranking results with the user preferences and recommended movies to the users. An affective recommender framework was proposed to provide personalized movie recommendations (Sivakumar et al., 2015) using audio-visual descriptors and connotations to offer the viewers’ emotional state. They adopted an SVR to predict the connotative values of each movie at the regression stage, and then the film nearing each other in the created connotative space were recommended to reviewers.

#### Clustering algorithms

The basic idea of video recommendations using a clustering algorithm is to cluster viewers or videos into groups based on the emotional similarity of viewers or the similarity of video features. The former recommends videos to users with similar emotional states, and the latter recommends unseen videos in the same cluster. In Niu et al. (2013), they presented a video browsing system called Affivir that dynamically adjusted session parameters according to viewers’ current mood by modeling user-watching behavior. For a given user, Affivir first analyzed the user’s emotional interest through an interactive process where user behavior of watching and skipping was recorded. When the user’s preference was learned, the unseen videos with similar affective responses based on affective similarities were recommended. Four affective video features generated identical videos. To improve the efficiency of video retrieval, videos in the database were pre-clustered based on video similarities. Subsequently, Niu et al. (2016) proposed an improved similarity calculation method, normalized validity—approximate graphs (NVAG), and adopted the block-based color histogram for similarity measurement. NVAG significantly improved the recommendation effect in video sharing compared with the Affivir algorithm.

#### AdaBoost learning algorithms

**FIGURE 3:**
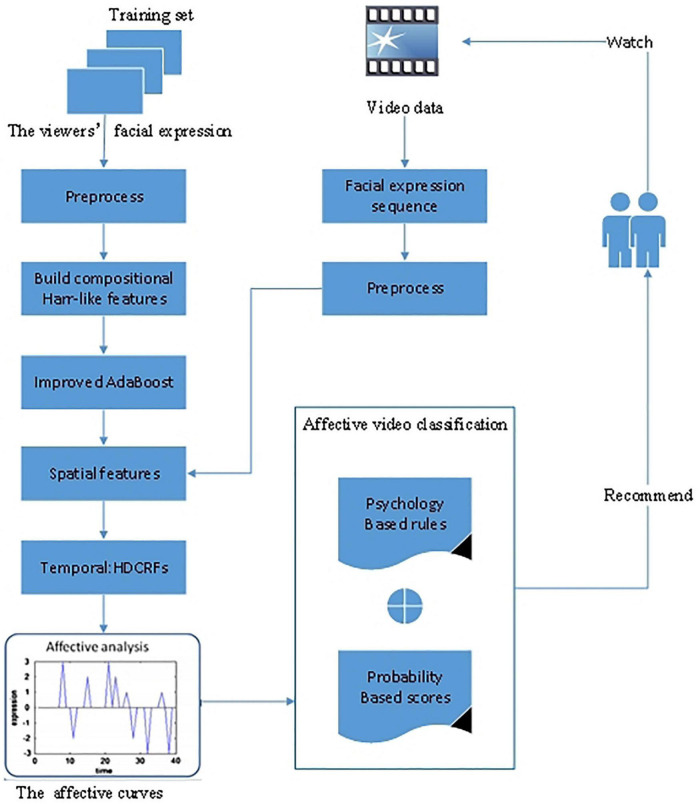
The framework of the improved AdaBoost learning algorithm (Zhao et al., 2013).

The core idea of adopting AdaBoost learning algorithms is selecting discriminative features to construct a facial expression classifier. Unlike the original AdaBoost algorithms selecting the best features in several rounds and generating a weak classifier, the AdaBoost algorithms used in facial expression tend to develop a mid-strong classifier based on a compositional feature. In Shu et al. (2018a), an AdaBoost classifier was used based on ECG signals obtained by a wearable device to analyze the emotional state, whether positive or negative. In Zhao et al. (2013), they proposed an improved AdaBoost learning algorithm to classify and recommend videos. The proposed method was based on facial expression recognition fused with spatiotemporal features. The spatial features combined Haar-like elements with training a mid-classifier and then were embedded into the improved AdaBoost learning algorithm to achieve spatial characteristics. For the temporal feature combination process, a time dimension variable was employed by the hidden dynamic conditional random fields (HDCRFs), and then the spatial features were embedded into HDCRFs to recognize facial expressions. The affective curve reflected the process of emotional changes. The video affection was classified into affective sections by psychology-based rules and probability-based scores by segmenting different emotional states. Finally, the videos were recommended to the users according to their affection states. Figure 3 illustrates the framework of the improved AdaBoost learning algorithm.

#### Matrix-based algorithms (MA)

The main idea of the matrix-based algorithms is to compile the multidimensional attributes in the data into a neural decision matrix (including the user’s emotional state, physiological parameters, etc.) and then conduct multiple standard neural analyses based on the neural decision matrix. To solve the recommendation problem of real estate advertising, video (Kaklauskas et al., 2018) considered the emotional state of buyers and proposed a neuro decision matrix based on house attributes, the emotional conditions of buyers, and physiological parameters. They selected the most personalized video alternatives according to the performance of a multiple criteria neuro analysis. They designed the neuro advertising property video recommendation system to provide effective video advertising for real estate buyers for a long time. In Dnodxvndv et al. (2018), they proposed a video neuro-advertising recommender model to analyze consumers’ emotions, measure the engagement of relevant ads, and make advertisements more efficient. The video neuro-advertising recommender model contained two Video Neuro-advertising Models and Systems (VINERS) Sub-models. The first Sub-model was based on the compiled neuro-matrix for assessing the effectiveness of a recommended advertisement; another Sub-model was used to generate a large number of variants for every viewer of an already developed advertisement.

#### Collaborative filtering (CF)

The general idea of AVRS research based on CF is mainly realized by measuring similarity, either recommending videos with similar emotions according to users’ emotional states or adding affective analysis factors when measuring users’ similarity. The collaborative filtering-based algorithm was one of recommender families’ most extensively used methods. In Soleymani et al. (2009), they proposed a collaborative, personalized affective video retrieval, which can retrieve videos according to emotional queries, arousal, and valence. Based on the traditional CF algorithm, Winoto and Tang (2010) considered the emotional factors and analyzed the impact between the user’s mood and the ratings of different movies. For example, whether a user with positive mood scores higher on romantic comedies or whether the user will score higher on action movies when he is in a tense mood.

Traditional CF algorithms recommend users based on their historical behavior similarity. However, new users face the cold-start problem. Instead of using historical behavior records (Choi et al., 2016), the changes in users’ facial scales were used to describe the dynamic preferences of usage. Through this method, they provided accurate, personalized recommendations for new and existing users, thus solving the users’ cold-start problem. In Diaz et al. (2018), they designed a recommender entirely based on the impression data of viewers. When a user views a video, the recommender system retrieves the metric data from user information. The video impression metric was used to determine which video resembled the metric of the current video. They tested three categories, i.e., the joy impression, the fear impression, and the sad impression. This impression-based recommender system was proved to break the lack of feature-based recommender systems.

#### Content-based filtering (CBF)

The dominant thought behind the CBF of AVRS is to incorporate affective video metadata, explicit feedback information, and user mood as part of an item or user attributes. In Canini et al. (2013), they believed that emotional content recommendations could better meet users’ tastes and preferences, so they extracted video tags and audio-visual features to combine semantic and affective video information. This method solved the problem of insufficient individual user preference space characteristics by processing user logs and boosting strategies. In Tkalčič et al. (2013b), a new database named LDOS-PerAff-1 Corpus was collected. To confirm the value of the new database consisting of emotion tags and the users’ ratings, they used four recommendation algorithms for verification: a fusion content-based algorithm, a collaborative filtering algorithm, an emotion detection algorithm, and matrix factorization. These four algorithms were tested involving different values of the used corpus in the recommendation, including the effectiveness of affected data improving the content-based advice, personality information that improves the cold-start problem, the role of emotion detection methods in face recognition, and user preferences for items with different favorite attributes.

Video data on the Internet does not exist alone but co-exist. For example, multimedia resources can contain video, audio, images, and other forms of existence at the same time (Soleymani et al., 2015). The affective analysis of multimedia content focuses on estimating users’ expected emotional state. In Deldjoo et al. (2018), they developed a content-based multimedia recommendation system (CB-MMRS) model based on CBF according to distinct resources. For video recommendation, items came from videos, movies, movie clips, trailers, etc. Items were used to match the user’s emotional state and obtain clear feedback by stimulating the user’s emotional state or by analyzing multimedia data.

#### Knowledge graph (KG)

**FIGURE 4:**
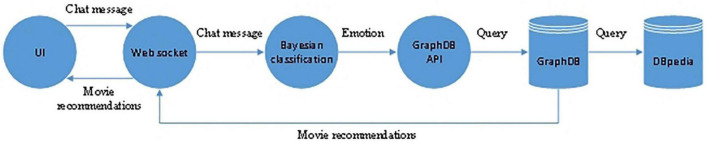
The recommendation process based on KG (Breitfuss et al., 2021).

The central idea behind KG of AVRS is to look for a particular emotion by KG, which has a similar emotion state extracted from user movie reviews. In Qi et al. (2021), they aimed to choose a small set of video frames based on the viewers’ personalized interest for video highlight detection. Specifically, they extracted the concept representation video clips by a front-end network, the concepts were used to build an emotion-related KG, and the relationships in the graph were related to the external public KGs. The emotional state influences decision-making when users consume movies. Therefore, a knowledge graph-based method (Breitfuss et al., 2021) was proposed to include the emotional state factor in movie recommendations. They extracted emotions from pre-existing movie reviews to construct the knowledge graph. To test the efficiency of the proposed method, they developed a chatbot with a reasoning mechanism combing users’ emotions analyzed from chat messages. Figure 4 shows the recommendation process based on KG. The chat messages of movie reviews between an AI chatbot and a user was extracted and categorized by a Bayesian classifier based on emotions. Natural language processing technology was used to remove emotions. To promote the speed of data retrieval, a graph database named graph DB API was employed to store the processing emotions.

#### Genetic algorithms (GA)

The GA is often used to solve the optimization problem of multiple objectives with conflicts. In AVRS, the critical idea of GAs is to balance the imbalance between users’ emotional preferences and actual business objectives. In Yadati et al. (2014), they studied the application of emotion analysis in in-stream video advertising as one of few excellent video recommendation works based on affective analysis and considering multiple objectives. They explained that emotion played a vital role in users’ purchasing behavior, and the consideration of emotional influence should be added to video advertising. Therefore, they proposed a method of Computational Affective Video-in-Video Advertising (CAVVA) strategy, which mainly considered two factors: identifying candidate advertising insertion points and the most appropriate advertisement. They modeled the problem as non-linear integer programming. Due to the conflict between these two objectives, minimizing the impact of advertising insertion on users and maximizing users’ participation in advertising, they adopted a genetic algorithm to solve the above conflict problems.

#### Hybrid recommender systems (HRS)

The dominant thought of employing HRS in AVRS is that combining multiple algorithms involving the viewers’ emotional states can promote recommendation efficiency. The effect of video recommendation by a single algorithm is limited, so researchers turned to HRS. In Wakil et al. (2015), they provided a hybrid model combining CF, CBF, and emotion detection algorithms. The CF and CBF algorithm was used to capture users’ preferences, and the emotion detection algorithm considered the influence of users’ emotion, which the traditional recommendation algorithms did not consider. An exciting research direction on video recommendation is temporary saliency, i.e., detecting the most critical video events, which may be the most attractive parts for users. A time series of arousal model (Mugellini et al., 2014) was designed based on audio-visual features to analyze users’ emotions. The multimodal system helps extract the parts that users may be interested in and can combine with various recommendation algorithms.

To summarize, in the last few years, researchers have made great efforts to video retrieve and recommendation domains by various traditional recommendation algorithms, including SVM/SVR, clustering, AdaBoost, MA, CF, CBF, KG, GA, and HRS. Some of their research works have achieved remarkable success, promoted the progress of AVRS, and improved the efficiency and quality of viewers’ access to video information. However, these algorithms still face the following problems:

1) Although the algorithm is simple and easy to implement, it cannot make accurate judgments on complex scenarios, and the recommendation effect is minimal. For example, Niu et al. (2013) recommended videos by clustering viewers’ moods, which was not a personalized recommendation strategy, and thus the recommendations may not work well.
2) The experiment databases are relatively small and not diverse. The portability of the recommendation strategy generated based on such a database is low, significant-good results on one database, while probably inferior on other databases. For example, Zhao et al. (2013) relied heavily on exaggerated and unnatural facial emotion expressions and lacked direct and intuitive expression, making the recommendation model unsuitable for the actual situation.

### Deep learning-based methods

Traditional recommendation algorithms, such as matrix factorization algorithms, are linear models, and the recommended effect is limited. Compared with conventional linear recommendation models, deep learning (DL) (Zhang et al., 2019) can obtain the non-linear characteristics of user interaction data, thereby capturing more complex information about user interaction patterns (Dai, 2021). The sequential modeling of DL also shows promising aspects in processing speech recognition, text analysis, etc. Therefore, the recommendation effectiveness of deep learning in recommender systems has been superior. Deep learning has penetrated a series of fields; the publication of deep learning algorithms has grown exponentially in industry and academia. Although DL has proved its essential role in the recommendation system, the exploration of the recommendation system in video recommendation is still limited, which needs to be paid attention by more scholars and supported by works in more fields. This subsection introduces several state-of-the-art DL models for solving affective video recommendations.

#### Reinforcement learning (RL)

**FIGURE 5:**
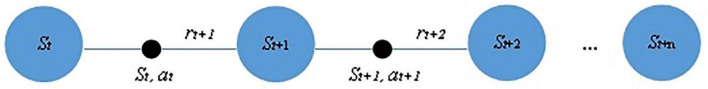
The RL sequence of states and actions (Tripathi et al., 2018).

The core idea of adopting RL in AVRS is that DL can continuously and dynamically learn strategies through the real-time state changes caused by the impact of users on the surrounding environment to maximize the cumulative reward. In Leite et al. (2022), they discussed the role of deep reinforcement learning (DRL) in video recommendation when used in a virtual learning environment. They also considered two different student groups, i.e., common effect and high effect. They designed a recommender system including five categories, i.e., the new videos to watch, the students communicating the current topic with a new tutor, the students displaying the segment with the current tutor, the corresponding piece with a new tutor, and the following video to watch. The type of recommender system was determined by the scores of students’ tests and the sensor-free participation detection model. The recommended strategy was based on a DRL algorithm. It was evaluated by a large field experiment, which showed the effectiveness of video recommendations during the regular school period. In Tripathi et al. (2018), they believe that the cognitive preferences of viewers are dynamic and should track the behavior of viewers and their cognitive preferences for different emotions in real-time. Therefore, they proposed an RL method to learn video recommendation decisions and monitor the interaction between users and recommended videos in real-time through the created user interface and webcam. Figure 5 illustrates the RL sequence of states and actions. The *S*_*t*_, *a*_*t*_, and *r*_*t*_ demonstrate the state, action, and the reward of time *t*, whereas *r*_*t*+1_ represents the reward gained by performing action in the state of *s*_*t*_. The learning process continued until state *s*_*t*+_*_*n*_*.

#### Convolutional neural network (CNN)

The basic idea of CNN in affective video analysis is that the CNNs can be employed for feature extraction from various types of signals and information. In Hewitt and Gunes (2018), they deployed a CNN model for facial affective analysis used on mobile devices. The proposed CNN model incorporates three variants of CNN architectures (i.e., AlexNet Variant, VGGNet Variant, and MobileNet Variant), which consider both the high performance and the low storage requirements. In Kwon et al. (2018), they designed a CNN architecture for accurate emotional classification. The CNN model extracts both temporal and frequency characteristic features from electroencephalogram signals and the pre-processed galvanic skin response (GSR) signals. The electroencephalogram signals reflect temporal characteristics as human emotions are time sequence data. A wavelet transform represents the frequency feature through the frequency axis. In Yang et al. (2019), they presented a multi-column CNN model using EEG signals for emotion recognition. The decision of the proposed CNN model is generated by a weighted sum of multiple individual recognizing modules.

**FIGURE 6:**
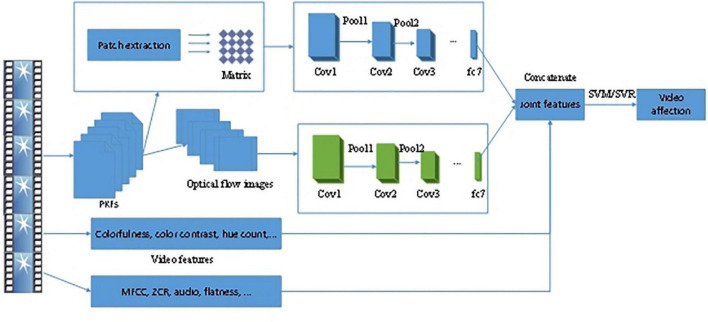
The framework of the proposed method (Zhu et al., 2019).

Unlike the above method of detecting the viewer’s emotion change through the device, Zhu et al. (2019) automatically recognized the viewer’s emotion by acquiring the information about the protagonist. They used a protagonist-based key frame selection strategy to extract features from video clips to alleviate the considerable workload of analyzing a large amount of video information. Then, the characteristics of keywords were fed into a CNN model based on optical flow images, and the CNN model incorporated temporal information from video clips. Then all of the features were fused as inputs of an SVM and SVR model for affective video recognition. The framework of the proposed method (Zhu et al., 2019) is shown in Figure 6. The framework is composed of two parts: feature extraction and feature concatenation. In the first process, they employed two CNN models to extract features related to hand-crafted visual and audio elements. The protagonists’ keyframes (PKFs) were selected from video clips. Then, two parallel extraction strategies were adopted to collect the matrix and optical flow images through two CNN models. These features were finally concatenated to map the affective dimension by an SVM/SVR model.

#### Long short-term memory (LSTM)

The dominant thought of adopting LSTM models in emotional video classification is that LSTMs can consider temporal, spatial, and frequency characteristics of various signals and information. In Alhagry (2017), an LSTM is adopted to learn the EEG features for emotional video recognition. The LSTM model takes the dense layer to classify the raw EEG features into low and high arousal, valence, and predicting the continuous scale between 1 and 9. In Wang et al. (2020), they established a Bi-LSTM model to extract emotional features for analyzing danmaku video data and users’ affective characteristics. The Bi-LSTM model classifies the users’ emotions into four dimensions, i.e., pleasure, anger, sorrow, and joy. In Zhang and Zhang (2017), they studied the inherent correlations between video content and the viewers’ affective states by presenting an LSTM model, which simultaneously predicts the arousal and valance dimensions. The LSTM model extracts a collection of low-level multimodal features from videos and projects these features into arousal and valence value pairs. In Nie et al. (2020), they considered the relations between the utterances and handled the multimodal feature fusion problem in the feature learning process with an LSTM-based model. In Ogawa et al. (2018), they introduced a Bi-LSTM network, which collaboratively adopts video features and EEG signals. They first used transfer learning for video classification as the limited number of video labels which difficult to classify. Then, a user study was conducted to verify the effective representation of EEG signals calculated by Bi-LSTM.

In Cao et al. (2022), they proposed the Visual Enhanced Comments Emotion Recognition Model (VECERM) to analyze users’ emotions, thereby overcoming the problem of user-generated comments related to plots. The VECERM model was composed of four layers.

##### Input embedding layer

In the input embedding layer, two significant parts are included: users’ text data comments and the images of video frames. This layer reduces the dimension of the input information, VGG processes the video information, and the Transformer processes the text information. The Transformer then converts the text representation into embedding vectors.

##### Context enhancement layer

Since text information and comments are synchronized, the Context Enhancement Layer mixes video information and text data through the attention mechanism.

##### Emotion attention layer

The purpose of the Emotion Attention Layer is to mine the emotional semantics of the comment text to obtain a good text representation. Due to the short length of the text, Bi-directional Long Short-Term Memory (BiLSTM) is adopted for mining the text data.

##### Classification layer

The Classification Layer realizes the classification of users’ emotions throughout the whole connection layer. This is a multi-classification classification problem, including glad, dismissed, sad, amazed, and afraid.

**FIGURE 7:**
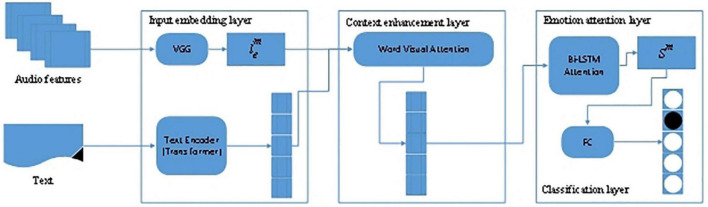
The architecture of VECERM (Cao et al., 2022).

The VECERM architecture is shown in Figure 7.

#### Multilayer perception (MLP)

The central idea behind the MLP of AVRS is to extract features from multimodal data to classify emotional expressions, e.g., visual, audio, and textual information. Krishnamurthy (2020) utilized an MLP network to classify user sentiments. The MLP model analyzes the users’ emotions based on web recordings from multimodal resources. They employed a feature-level fusion method to fuse the extracted features from various modalities, i.e., video, posts, and pictures. An oppositional grass bee algorithm then chooses the extracted features to generate the best optimal feature set. In Boughrara et al. (2016), they proposed an MLP for facial expression classification. The established MLP model consists of a single hidden layer, which seeks to find synthesis parameters in the training stage. They adopted a biological vision-based facial description in the feature extraction step to extract face image features.

**FIGURE 8:**
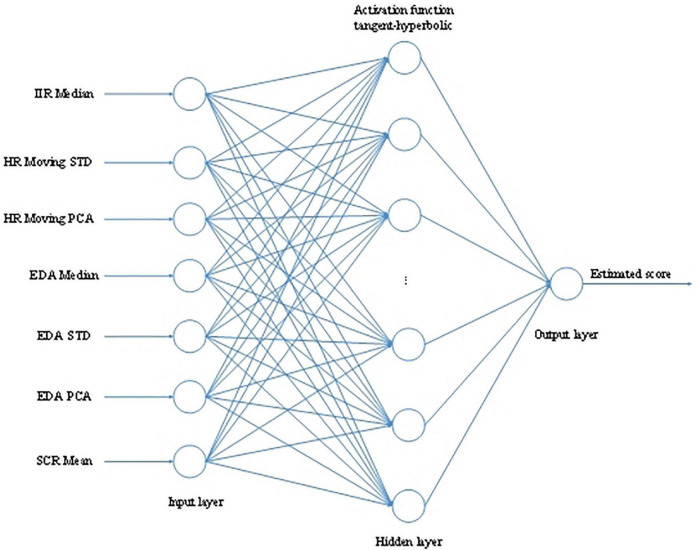
The MLP model (Ðorđević Čegar et al., 2020).

To predict the emotional state of users when watching a stereoscopic 3D video, Ðorđević Čegar et al. (2020) extracted features from the volunteers’ psychological data of ECG, EDA, and EEG signals and then used an emotional state estimator based on feedforward multilayer perception artificial neural network to predict the state of viewers when they were viewing different kinds of stereoscopic 3D video content. The MLP model is shown in Figure 8. The configuration of MLP based on HR and EDA selected features were as the input features, including IIR Median, HR Moving STD, HR Moving PCA, EDA Median, EDA STD, EDA PCA, and SCR Mean. They adopted the Levenberg-Marquardt back-propagation algorithm for training the network. The output of MLP was a linear activation function, which generated the estimated scores.

#### Deep hybrid models (DHM)

The fundamental idea of implementing DHM is combining different DL models (e.g., CNN, RNN, LSTM, RL, etc.). The fusing mode of multiple DL models can be either the output of one or several models is used as the input of another model, or several models simultaneously extract the features of video or multimodal data or signals. The combination of several models improves the limited non-linear performance of a single model (e.g., LSTM has a memory for long-time data processing). In Zhang et al. (2018), they established an audio-visual emotion recognition model, which is fused with a CNN, 3D-CNN, and a Deep Believe Networks (DBNs). The designed model is a two-step procedure. The CNN and 3D-CNN are firstly pre-trained according to a large-scale of both image and video tasks, which are fine-tuned to learn audio and visual segment features. Then, the output of the former step is combined into a fusion network to build a DBN model, and a linear SVM obtains the final results of emotional classification. In Fan et al. (2016), they proposed a hybrid DL model for video-based emotional recognition. The model is the combination of a recurrent neural network (RNN) and a 3D CNN. The 3D CNN models the video appearance and motion concurrently, while the RNN model processes the appearance features obtained by the CNN model over individual video frames, which are used for the input features, then RNN encodes the motion. In Yenter (2017), they produced an architecture that combined CNN and LSTM models for textual sentiment analysis. The CNN model is consisted of multiple branches, whereas the LSTM model is a word-level classification. The output of CNN branches is transferred to the LSTM and then concatenated to a fully-connected layer to generate a single output for sentiment polarity classification.

**FIGURE 9:**
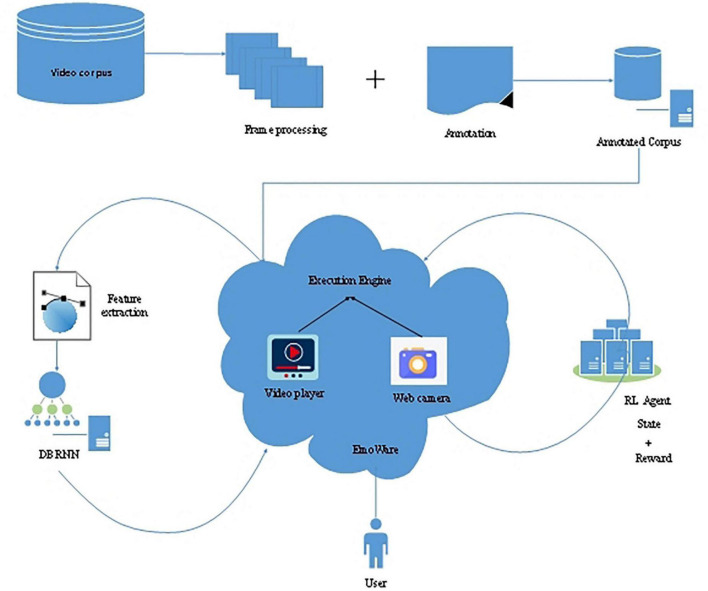
The framework of EmoWare (Tripathi et al., 2019).

Mishra et al. (2020) established a fascinating empirical analysis. Firstly, they used two CNN models (AlexNet and GoogLeNet) and an LSTM model to classify EEG data into different emotion categories. The purpose was to recognize the emotional state of EEG data through the deep learning model. Using the pre-trained CNN and LSTM models can reduce the computing cost of the training network through simple parameter adjustment. Then, these models were used to verify whether the trained models were universal and effective in different fields. In Liu et al. (2017), they presented two attention mechanisms, i.e., LSTM and RNN, for emotion recognition. These two models integrate temporal attention and band attention, which are based on untrimmed visual signals and EEG signals. The LSTM and RNN models take all the signal data as inputs and then generate representations of each signal, which are transferred to a multimodal fusion unit for predicting the emotional labels. Tripathi et al. (2019) designed a personalized and emotional intelligence video recommendation engine named EmoWare, which employed reinforcement learning (RL) and deep-bidirectional recurrent neural networks (DBRNN) models. The framework of EmoWare is shown in Figure 9.

To summarize, deep learning-based AVRS algorithms can learn the potential characteristics of audio, text, video, and other multimedia and obtain representations and abstraction from multiple levels, resulting in its significant advantages in dealing with emotional analysis. For example, the CNN can capture the global and local features and analyze spatial information changes during short time periods of video clips, remarkably enhancing efficiency (Fonnegra, 2018). The RNN architecture is good at processing sequential data by remembering former computations in loops. Each deep learning algorithm has its personalized advantages and disadvantages. Therefore, researchers combine several deep learning models to solve complex problems. Especially, Tripathi et al. (2019) adopted RNN and LSTM algorithms concurrently. However, deep learning is still in its infancy in affective video recommendation. The research work of exploration is scarce, and the available databases are also very precious. It still needs a large amount of research support.

## Affective video recommendation databases

In this section, we introduce the existing 31 valuable databases which play a vital role in AVRS research. These databases are composed of multiple modes, including comments, ratings, videos, films, audio, images, etc. There are various methods to obtain these data, such as capturing the changes in the viewer’s facial expressions through webcams, getting the user’s physiological signals through EEG, questionnaires, or a combination of these methods. Most of these databases are manually collected by researchers, which is time-consuming and error-prone. In Lucey (2012), they provided an effective way to construct two databases without manually scanning the full movies, and the movie labelers only reviewed video clips recommended by an RS. These video clips are the most representative. This method can quickly collect and obtain much-annotated video information. The various databases and their details are listed in Table 4.

**TABLE 4:** The databases for affective computing.

Name	Details	Publication
The affective feedback database	Questionnaires of 24 participants on tasks, search process, and emotional experience of the information-seeking process	Arapakis et al., 2009a
Cohn–Kanada expression database	The database has 2105 digitized image sequences of 182 adult subjects, suitable for comparative studies by multiple tokens of most primary FACS action units.	Zhao et al., 2011
Moviepilot mood track	It consists of 4.5M ratings assigned by 105K users on 25K movies. Various contextual information is provided, i.e., gender, age, production year, the audience of each movie, movie-mood tag, etc.	Shi et al., 2013
The Hollywood movie video clips database	Contains 155 video clips from Hollywood movies, annotated by 40 participants with more than 1,300 annotations.	Soleymani et al., 2009
The Tellyads and YouTube video clips database	Contains 15 videos of 165 min duration from various genres, e.g., TV shows, movie clips, and news broadcasts.	Yadati et al., 2014
The affective property movie database	The database contains more than 2,000 videos; movie affective properties are measured by arousal and valence.	Niu et al., 2013
Nvidia 3D Vision database	The database contains nine stereoscopic sequences of nearly 2 min duration.	Ðorđević Čegar et al., 2020
The movie profile database	It contains an item profile of various attributes describing the movie content.	Wakil et al., 2015
The five emotional reactions database	Two standard webcams are operating in real-time used to capture the users’ facial expressions and estimate the pulse. The users’ reactions can be classified into five categories: happiness, sadness, anger, fear, and surprise.	Bohlin et al., 2019; Soni et al., 2019
Cohn–Kanada database	Consists of 100 students of different races, i.e., African–American, Asian, and Latino. Each subject performs a series of 23 facial displays. The selected sequences are labeled with six emotions: anger, disgust, fear, happiness, sadness, and surprise.	Zhao et al., 2013
The clicker and emotional reaction database	It consists of 30 subjects from the age of 18–35. Each subject watches five videos, and two webcams monitor the behavior. The issues must also be surveyed according to their watching and rating.	Diaz et al., 2018
DEAP	The database is a multimodal database using EEG and physiological signals for emotion analysis. The database obtains 32 subjects’ 1-min musical physiological video signals.	Soleymani et al., 2012; Dabas et al., 2018; Mishra et al., 2020
Algebra video field test database	The data are collected by a field experiment of 18,925 school students and 152 teachers in 149 schools.	Leite et al., 2022
Cohn Kanade database	It contains photos of different emotions, from a neutral state to an explicit one.	Leite et al., 2022
The 0-MOOD, 7-MOOD, 16-MOOD	It contains 0, 7, and 16 mood states, respectively.	Winoto and Tang, 2010
The user action session database	Affivir constantly crawls video data from the Internet, and user preference features are extracted.	Niu et al., 2013; Niu et al., 2016
The format video database	It contains 1,000 format mp4 videos ranging from 30 s to 10 min. The videos are from various websites, i.e., Youku.com, YouTube.com, etc.	Niu et al., 2017
The footwear advertising videos database	The user facial features and ratings of 52 subjects record the movement of vital facial points continuously.	Choi et al., 2016
The NEAR database	The NEAR database consists of a wide range of databases, i.e., the Property Video Clip Ads Database, a text database of video clips.	Kaklauskas et al., 2018
LIRIS-ACCEDE	It contains 160 feature films and short films from 9,800 video clips. It is the largest video database with emotional labels and can be used for video indexing, summarization, and browsing.	Baveye et al., 2013; Baveye et al., 2015; Zhu et al., 2019
PM-SZU	It is a new database for affective video analysis. It consists of 386 video clips extracted from 8 films.	Zhu et al., 2019
The metractitic.com and imdb.com database	It consists of 2,627,476 movie reviews.	Breitfuss et al., 2021
Danmu database	It contains a large amount of user-generated comments from Bilibili.	Cao et al., 2022
LDOS-PerAff-1 Corpus	It consists of subjects’ affective responses to video clips, answers are annotated in the continuous valence-arousal-dominance space, and topics are annotated with personality information.	Tkalčič et al., 2011a,2013b
Mechanical Turk setup	It contains affective annotations for the corpus to evaluate viewers’ reported boredom.	Martha and Larson, 2013; Soleymani et al., 2014
Multidimensional sentiment dictionary from Ren CE	It includes 1,487 blogs and many emotional words and is labeled as a vector of 8 dimensions.	Pan et al., 2020
YouTube video clips	Containing f 600 videos, 480 had transcripts.	Pan et al., 2020
LDOS-CoMoDa	It consists of contextual information and ratings on the users’ consumed movies and personality profiles.	Odic et al., 2014
The IMDB movie scenes	Some 240 users are viewing videos on 25 movie scenes on IMDB. The duration is recorded.	Benini et al., 2011
The AFEW database	A dynamic, temporal facial-expression data corpus contains short video clips of facial expressions close to the real world.	Lucey, 2012
The SFEW database	It is a static, harsh conditions database consisting of seven facial expression classes.	Lucey, 2012

## The audience responses

The audience response to a video can be obtained in various ways, mainly including two categories: explicit acquisition and implicit acquisition. Standard methods for explicit acquisition include user interactions (i.e., watching videos, skipping videos.), questionnaires, surveys, and quizzes. The questionnaires can be achieved through self-assessment manikin (SAM) (Dnodxvndv et al., 2018). There is a wide range of ways to implicitly obtain the emotional characteristics of viewers, including facial expressions or features, measuring skin estimated pulse, heart rate, body gestures, reviews, or comments. The viewers’ psychophysiological signals of heart rate (HR) are calculated from an echocardiogram (ECG) (Baveye et al., 2015), while electro-dermal activity (EDA) and brain activity (BA) are from electroencephalography (EEG) signals (Ðorđević Čegar et al., 2020). The facial expressions or features [e.g., gaze distance (Soleymani and Pantic, 2012)] can be obtained by a camera (Tkalčič et al., 2013a). The questionnaire can accurately convey the emotional state of users. However, it is also faced with the problem that the amount of data is limited, affecting the viewing experience, costly for organizations to conduct, and volunteers sacrifice much time (Mulholland et al., 2017). Therefore, an implicit acquisition that obtains affective states from face recognition, heart rate, mood, EDA, BA, and body gestures plays a significant role and provides more ways for affective video recommendation. The method of implicit acquisition is more flexible. Only by recording the physical signs of the viewer can we obtain the emotional state through the algorithm. Martha and Larson (2013) provide a unique perspective to analyze the emotional states, that is, perceived connotative properties, which prove to be more intersubjectively shared.

Table 5 shows the audience responses in different publications. It can be inferred that facial expressions/features, skin-estimated pulse/heart rate, movie reviews/comments, and questionnaire/survey/quizzes are the most frequently used user responses in affective video computing. Some researchers also get users’ emotional feedback on videos from other different perspectives, such as mood (Winoto and Tang, 2010), EDA (Ðorđević Čegar et al., 2020), BA (Ðorđević Čegar et al., 2020), body gestures (Hassib et al., 2017), and perceived connotative properties (Martha and Larson, 2013). Some experimental studies use one of these methods to obtain emotional expression, but most of the research work uses a combination of multiple user feedback methods. For example, Bohlin et al. (2019) and Soni et al. (2019) evaluate the emotional state by facial expressions/features and skin-estimated pulse/heart rate (Diaz et al., 2018) adopt the method of combination of skin-estimated pulse/heart rate and questionnaire.

**TABLE 5:** The audience responses in different publications.

Audience responses	Publications
Facial expressions/features	Soleymani and Pantic, 2012; Zhao et al., 2013; Boughrara et al., 2016; Choi et al., 2016; Kaklauskas et al., 2016; Mahata et al., 2017; Diaz et al., 2018; Fonnegra, 2018; Hewitt and Gunes, 2018; Kaklauskas et al., 2018; Bohlin et al., 2019; Soni et al., 2019; De Pessemier et al., 2020; Mishra et al., 2020; Leite et al., 2022
Skin-estimated pulse/heart rate	Dabas et al., 2018; Diaz et al., 2018; Shu et al., 2018a; Bohlin et al., 2019; Soni et al., 2019; Ðorđević Čegar et al., 2020
Mood	Winoto and Tang, 2010
EDA	Ðorđević Čegar et al., 2020
BA	Alhagry, 2017; Liu et al., 2017; Kwon et al., 2018; Ogawa et al., 2018; Yang et al., 2019; Ðorđević Čegar et al., 2020
User interactions	Niu et al., 2013; Niu et al., 2016
GSR	Kwon et al., 2018
Body gestures	Hassib et al., 2017
Perceived connotative properties	Martha and Larson, 2013; Zhang and Zhang, 2017
Movie reviews/comments/web recordings	Mulholland et al., 2017; Yenter, 2017; Tripathi et al., 2019; Krishnamurthy, 2020; Pan et al., 2020; Wang et al., 2020; Breitfuss et al., 2021; Cao et al., 2022
Questionnaire/survey/quiz	Arapakis et al., 2009a; Soleymani and Pantic, 2012; Tkalčič et al., 2013b,^2014^; Polignano, 2015; Hassib et al., 2017; Diaz et al., 2018; Dnodxvndv et al., 2018; Kaklauskas et al., 2018; Bohlin et al., 2019; Zhu et al., 2019; Mishra et al., 2020; Kaklauskas et al., 2020; Leite et al., 2022

## Evaluation methods

The commonly used performance indicators include mean accuracy, precision/recall/F1, mean absolute error (MAE), mean square error (MSE)/root mean square error (RMSE), confusion matrix, and valence, arousal, and dominance. However, viewers do not need perfect prediction accuracy but need wise recommendation strategies. Therefore, in addition to the former metrics, several researchers also began to pay attention to the quality of perceived recommendations to evaluate their models and algorithms. For example, Arapakis et al. (2009a) adopted Pearson’s ChiSquare test and the Dependent *t*-test to analyze the emotion variance and the recommender system’s performance. Niu et al. (2013, 2016) used CTR, session length, and points test to evaluate the recommendation performance. The higher the CTR, the longer the session length, and the better the recommendation quality. The compiler average causal effect (CACE) evaluator was employed by Leite et al. (2022) to test the impact of recommendations offered to the treatment group. Breitfuss et al. (2021) tested their knowledge graph-based recommendation strategy by various metrics, including Sparsity impact, the granularity of emotions, extensibility, recommendation quality, and additional characteristics. Table 6 lists the evaluation metrics used in different publications.

**TABLE 6:** The evaluation metrics of different publications.

Metrics	Related research papers
Pearson’s chi-square test and the dependent *t*-test	Arapakis et al., 2009a
Mean accuracy	Zhao et al., 2011, 2013; Tkalčič et al., 2013b; Fan et al., 2016; Alhagry, 2017; Liu et al., 2017; Yenter, 2017; Zhang and Zhang, 2017; Dabas et al., 2018; Fonnegra, 2018; Hewitt and Gunes, 2018; Kwon et al., 2018; Shu et al., 2018a; Zhang et al., 2018; Bohlin et al., 2019; Soni et al., 2019; Yang et al., 2019; De Pessemier et al., 2020; Krishnamurthy, 2020; Mishra et al., 2020; Nie et al., 2020; Wang et al., 2020; Qi et al., 2021; Leite et al., 2022
Precision/recall/F1	Niu et al., 2013; Shi et al., 2013; Liu et al., 2017; Ogawa et al., 2018; Zhang et al., 2018; Tripathi et al., 2019; Yang et al., 2019; Krishnamurthy, 2020; Mishra et al., 2020; Wang et al., 2020; Cao et al., 2022
MAE	Winoto and Tang, 2010; Choi et al., 2016
MSE/RMSE	Boughrara et al., 2016; Hewitt and Gunes, 2018; Tripathi et al., 2019; Zhu et al., 2019; Ðorđević Čegar et al., 2020
ROC	Winoto and Tang, 2010
CTR	Niu et al., 2013; Niu et al., 2016
Session length	Niu et al., 2013; Niu et al., 2016
Confusion matrix	Tkalčič et al., 2011b,2013b; Zhao et al., 2013; Boughrara et al., 2016; Fan et al., 2016
CACE	Leite et al., 2022
Sparsity impact, the granularity of emotions, extensibility, recommendation quality, additional characteristics	Breitfuss et al., 2021
Valence, arousal	Wang and Cheong, 2006; Soleymani et al., 2009; Soleymani and Pantic, 2012; Oliveira et al., 2013; Tkalčič et al., 2013b; Liu et al., 2017; Kwon et al., 2018; Yang et al., 2019

## Challenges and opportunities

In this survey, an overview of traditional recommendation methods (e.g., SVM, SVR, CF, CBF, AdaBoost, GA, Clustering, MA, KG, HRS) and deep learning-based technologies (e.g., CNN, MLP, RL, RNN, LSTM, DHM) adopted in AVRS has been depicted. The research of AVRS is challenging since a tremendous effort involving a multidisciplinary understanding of human behavior and perception and multimodal approaches integrating different modalities are required, such as text, audio, image, and video. Although many scholars have begun to pay attention to the field of AVRS in recent years and have made valuable contributions from the perspective of data, models, and algorithms, AVRS is still in its infancy. The challenges of the AVRS domain mainly come from the following three aspects:

(1) Insufficient data and data analysis is highly sophisticated.

Much of the existing facial data exists a lot of unnatural and exaggerated expressions (Zhang et al., 2022). More intuitive, natural, scalable, and transportable facial expressions are needed. In addition, the research on emotion analysis in the field of recommender systems is not comprehensive. More complex expressions that are not easily exposed should be paid attention to, for example, micro-expression recognition (Ben et al., 2021). Additionally, the EEG signals are difficult to analyze from which part of the brain the electrical activity originates (Dabas et al., 2018). This undoubtedly makes it more challenging to accurately diagnose users’ emotional states on video.

(2) Combining existing models and algorithms with deep learning-based techniques is insufficient.

The exploration of affective video recommendation algorithms based on deep learning is currently limited. It only involves several deep models, such as RL, CNN, RNN, LSTM, MLP, and hybrid algorithms of several models. More advanced works and better performance are needed based on emotional analysis recommendations. The state-of-the-art technologies emerging in recent years may also be combined with the AVRS domain, e.g., the self-attention-based transformer model in sentiment changes detection (Wu et al., 2020), and the generative adversarial network (GAN) may provide data augmentation for small-scale video or multimodal databases (Ma et al., 2022).

(3) The research direction is monotonous.

The current focus is limited to the accuracy of prediction on video recommendations, and the main problem to be solved is the cold-start or long-tail effect (Roy and Guntuku, 2016). However, other research directions of recommendation systems are not involved, such as multiobjective recommender systems (MORS) (Wang and Chen, 2021) or multi-task recommender systems (MTRS) (Ma et al., 2018) and explainable recommender systems (ERS) (Zhang and Chen, 2020). The MORS or MTRS can incorporate more objectives or tasks into the video recommendation based on affective computing; these models focus on more extensive aspects of recommendation quality, such as diversity, novelty, etc. The ERS is a promising research direction, which provides the viewers with the recommendation reasoning according to their facial expressions, body gestures, or other kinds of emotional responses.

## Author contributions

DW: conceptualization, methodology, formal analysis, resources, data curation, writing—original draft preparation, and visualization. XZ: validation and supervision. Both authors contributed to the writing—review and editing and approved the submitted version.
